# New advances in protein engineering for industrial applications: Key takeaways

**DOI:** 10.1515/biol-2022-0856

**Published:** 2024-06-17

**Authors:** Giles Obinna Ndochinwa, Qing-Yan Wang, Nkwachukwu Oziamara Okoro, Oyetugo Chioma Amadi, Tochukwu Nwamaka Nwagu, Chukwudi Innocent Nnamchi, Anene Nwabu Moneke, Arome Solomon Odiba

**Affiliations:** Department of Microbiology, Faculty of Biological Science, University of Nigeria, Nsukka, 410001, Nigeria; State Key Laboratory of Biomass Enzyme Technology, Guangxi Academy of Sciences, Nanning, Nanning, 530007, China; National Engineering Research Center for Non-Food Biorefinery, Guangxi Academy of Sciences, Nanning, Nanning, 530007, China; Department of Pharmaceutical and medicinal chemistry, Faculty of Pharmaceutical Sciences, University of Nigeria, Nsukka, 410001, Nigeria; Department of Genetics and Biotechnology, Faculty of Biological Sciences, University of Nigeria, Nsukka, 410001, Nigeria

**Keywords:** protein and enzyme engineering, industrial biotechnology, thermostability, *Escherichia coli*, *Saccharomyces cerevisiae*, yeast, bacteria, fungi, algae

## Abstract

Recent advancements in protein/enzyme engineering have enabled the production of a diverse array of high-value compounds in microbial systems with the potential for industrial applications. The goal of this review is to articulate some of the most recent protein engineering advances in bacteria, yeast, and other microbial systems to produce valuable substances. These high-value substances include α-farnesene, vitamin B12, fumaric acid, linalool, glucaric acid, carminic acid, mycosporine-like amino acids, patchoulol, orcinol glucoside, d-lactic acid, keratinase, α-glucanotransferases, β-glucosidase, seleno-methylselenocysteine, fatty acids, high-efficiency β-glucosidase enzymes, cellulase, β-carotene, physcion, and glucoamylase. Additionally, recent advances in enzyme engineering for enhancing thermostability will be discussed. These findings have the potential to revolutionize various industries, including biotechnology, food, pharmaceuticals, and biofuels.

## 1 Introduction

Gene manipulation involves the use of biotechnological techniques to alter the genetic material of an organism, enabling the production of proteins with desired characteristics [1]. Protein engineering has evolved over many decades (Table 1), and gene manipulation is no longer a technical challenge. For instance, human genes can be successfully integrated into the genome of mice, and plant genes can even be introduced into bacterial and yeast cells. One of the notable breakthroughs was the work of Stanley Cohen and Herbert Boyer, who transferred a human gene encoding insulin into bacterial cells to produce insulin [2]. This achievement highlights the universal nature of life’s basic instructions, enabling the decoding of genetic information from various organisms to produce proteins. In the area of agriculture, protein engineering facilitates the creation of genetically modified plants (GMOs) with unique properties. Typically, their fruits are characterized by increased durability, enhanced taste, stronger aroma, resistance to pests, and adverse environmental conditions. A notable example is Flavr Savr, a breed of genetically modified tomatoes resistant to rot due to a transformed gene responsible for the production of an enzyme that breaks down the cell wall [3]. Flavr Savr has been approved and is available for human consumption. Genetic manipulation has also been used to create modified animals with increased growth hormone levels, which quickly grow to large sizes, thereby reducing breeding costs in the long run [4].

**Table 1:** Major milestones in the progression of protein engineering

Year/decade	Milestone	Description	Reference
1953	Discovery of DNA structure by Watson and Crick	Laid the foundation for understanding the relationship between genes and proteins	[7]
1960s	Development of recombinant DNA technology	Enabled isolation, manipulation, and expression of specific genes, opening avenues for targeted protein modifications	[8]
	First successful protein engineering experiment	Anfinsen et al. refold and reactivated a denatured ribonuclease, demonstrating the possibility of manipulating protein structure and function	[9,10]
1970s	Development of site-directed mutagenesis	Introduced specific mutations into sequences, enabling targeted changes in protein function	[11]
	Emergence of rational design	Used structural information to predict the effects of mutations on protein structure and function, guiding the design of new proteins	[12,13]
1980s	Directed evolution	Mimicked natural selection in the lab, creating protein libraries with diverse mutations and selecting variants with desired properties	[14]
	Discovery of protein folding codes	Identified general principles governing protein folding, fueling efforts in *de novo* protein design	[15]
1990s	Development of computational tools for protein structure prediction and design	Advanced computing power and algorithms enabled more accurate predictions of protein structures	[16]
	First *de novo* designed proteins	Researchers successfully created functional proteins from scratch using computational design methods.	[17]
2000s	High-throughput screening	Automated platforms accelerated the identification of desired protein variants from large libraries	[18]
	Expansion of protein engineering applications	Extended beyond enzyme optimization into areas like biomaterials development, biosensors, and therapeutic protein design	[19,20]
2010s–Present	Machine learning and artificial intelligence integration	Advanced algorithms improved protein structure prediction, guided rational design, and facilitated protein function prediction	[21]
	Non-canonical amino acid incorporation	Expanded the building blocks of proteins, enabling novel functionalities	[22]
	Focus on protein–protein interactions	Engineering protein–protein interactions holds promise for new therapeutic strategies and functional materials	[23]
Looking forward	Continued advancements	Expected in computational tools, automation, and protein engineering techniques, accelerating the development of proteins with tailored properties for diverse applications	

Designing new proteins with desired functions is a complex task, but it can yield significant benefits to the pharmaceutical, biomedical, and other industries. While medical applications currently represent the most lucrative market for engineered protein products, synthetic enzymes are also utilized in the food industry for processing. Moreover, engineered enzymes have been employed in industries to detoxify pollutants such as plastics [5]. The vast sequence space and numerous structural constraints are the major factors that make designing improved proteins difficult. Factually, a protein composed of 100 amino acids can indeed have about 10^130^ possible variants, the vast majority of which are non-functional [6]. High-throughput biochemical assays and sequencing technologies have accelerated large-scale genetic screening, which is crucial for protein engineering. This has allowed for an in-depth exploration of the relationships between protein structure and function under conditions of controlled selective pressure. Such pressures could be external factors like temperature, pH, or substrate concentration, which are critical in the design and optimization of proteins for specific applications. This approach has significantly enhanced the understanding of protein evolution, adaptation, and other biological phenomena, paving the way for the development of novel proteins with desired properties.

**Figure 1:**
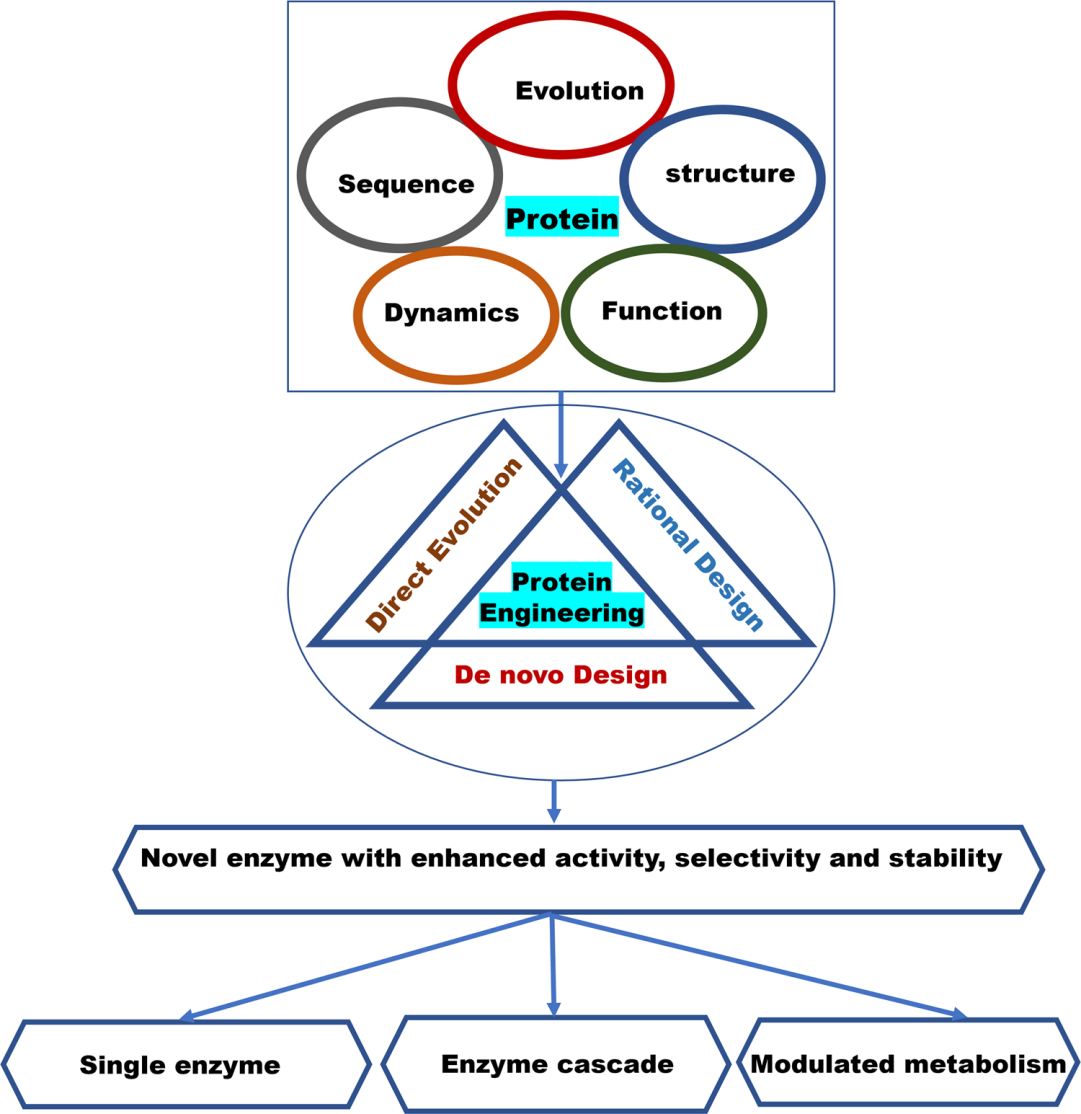
Overview of protein engineering for improved enzyme properties.

The fundamental principle of protein engineering involves modifying sequences through methods, such as direct evolution, rational design, and *de novo* design (Figure 1). These modifications aim to enhance the catalytic activity, stability, and selectivity, thereby improving the enzyme cascade system. Significant progress has been made in protein (enzyme) engineering methods, with current techniques showing notable improvements over traditional methods (Table 2). Emerging frontiers in enzyme immobilization, computational enzyme design, multi-enzyme systems, synthetic biology, and genome editing hold promise for further advancements in enzyme engineering. With continued research and innovation, enzyme engineering has the potential to revolutionize a wide range of industries, leading to a greener and more sustainable future.

**Table 2:** Comparison between the current protein engineering methods and traditional methods

	Traditional methods	New methods
Overview	Traditional protein engineering involves rational protein design and directed evolution. It often requires detailed knowledge of the structure and function of a protein	New advances leverage computational protein design to create new proteins and functions *in silico* [24]. They also use machine learning and artificial intelligence [25,26]
Advantages	Traditional methods are inexpensive and technically easy, as site-directed mutagenesis methods are well-developed	New methods accelerate the process, reduce costs, and enable more sophisticated engineering goals. They can also produce protein chains up to 164 amino acids long rapidly (Hartrampf et al., [27])
Limitations	The major drawback is that detailed structural knowledge of a protein is often unavailable. Even when available, it is very difficult to predict the effects of various mutations	While the sequence-conformation space that needs to be searched is large, the most challenging requirement for computational protein design is a fast yet accurate energy function
Applications	Traditional methods have been used to improve the function of many enzymes for industrial catalysis	New methods have been applied in the design of novel protein variants incorporating amino acids that do not occur naturally in cells [28]. They have also been used in the development of biocatalysts for industrial applications

To promote the industrial use of enzymes, several crucial factors must be considered, including catalytic efficiency, substrate specificity, and stability [26,29]. Catalytic efficiency is a fundamental component of enzyme engineering, where it decisively influences the rate and efficacy of enzymatic reactions [30,31]. In the field of industrial biotechnology, the need for highly efficient enzymes presents new challenges, particularly the necessity to significantly increase catalytic turnover (*k*_cat_) or efficiency (*k*_cat_/*K*_m_). Substrate specificity, on the other hand, is a crucial element in enzyme engineering. It refers to an enzyme’s capacity to selectively bind and catalyze a particular substrate over others. This specificity is dictated by the unique three-dimensional structure of the enzyme’s active site, which is specifically designed to accommodate certain substrates [32]. In an industrial context, this specificity ensures that engineered enzymes execute their intended reactions with efficiency and selectivity, thereby minimizing undesired side reactions. For example, in the pharmaceutical industry, enzymes exhibiting high substrate specificity are employed to synthesize drugs, resulting in fewer impurities. Similarly, stability against heat and organic solvents is particularly important due to the harsh conditions of industrial processes. Directed evolution has been instrumental in enhancing catalytic properties, and efforts have been made to create enzymes with new catalytic functions for non-natural reactions. Rational design, computational methods, and directed evolution have been combined to achieve this goal. Likewise, substrate channeling, an approach that directs intermediates to the next-stage enzymes, has been explored to enhance reaction rates and conversion yields in multi-enzyme processes [33]. Various strategies, including co-localization of enzymes and the use of scaffold molecules, have also been employed to facilitate substrate channeling and increase product yield.

In recent years, protein/enzyme engineering has seen numerous advancements, resulting in remarkable outcomes with potential for industrial application. This review aims to spotlight these recent achievements. It specifically explores the application of bacteria, yeasts, fungi, and algae systems in enzyme engineering. Additionally, it highlights recent bioengineering efforts to enhance thermostability, a critical factor for production efficiency [34]. Overall, this review intends to enrich the awareness of scientists, industries, and stakeholders about current developments in this very important field.

### 1.1 Metabolic engineering in *Escherichia coli* for the biosynthesis of specific compounds

*E. coli* is a versatile and well-studied bacterium that has been extensively employed in biotechnology for the production of various industrially important compounds. Recent advancements in metabolic engineering have further highlighted the versatility of *E. coli* in diverse bio-based chemical productions, leading to promising results in enhancing bio-products such as γ-aminobutyric acid (GABA), shikimate, d-allulose, monophosphoryl lipid A (MPL), caffeic acid, α-aminoadipic acid (AAA), lipoic acid (LA), l-arginine, erythritol, 2-phenylethanol, hydroxytyrosol (HT), 2′-fucosyllactose (2′-FL), *cis*-3-hydroxypipecolic acid (*cis*-3-HyPip), and various valuable compounds routed from heme biosynthesis using whole-cell biocatalysis. Yang et al. recently demonstrated the remarkable potential of *E. coli* in enhancing GABA production through a combination of enzyme evolution and metabolic engineering techniques [35]. By developing mutants of glutamate decarboxylase (GadBM4), a 20.27% increase in GABA productivity in one of the mutants (GadBM4-2) was achieved. Further improvement in GABA production was achieved by introducing the regulator GadE and enzymes from the pathway for the synthesis of pyridoxal 5′-phosphate. This resulted in a remarkable 24.92% increase and reached 76.70 g/L/h with high conversion efficiency. In a 5-L capacity bioreactor, the concentration of GABA in the fermentation broth reached an impressive 307.5 ± 5.94 g/L, with a production rate of 61.49 g/L/h when crude l-glutamic acid was used as the substrate. The versatility of *E. coli* extends to the production of shikimate, as demonstrated by Li et al. [36], where, using a comprehensive approach, they identified and utilized ten key target genes to promote metabolic flux towards shikimate biosynthesis [36]. The engineered *E. coli* strain, SA05, achieved a remarkable shikimate production of 78.4 g/L. Further optimization reduced unwanted by-products and enhanced shikimate productivity by 23.2%. In a 30-L fermenter, SA09 achieved a notable shikimate titer of 126.4 g/L, a glucose yield of 0.50 g/g, and 2.63 g/L/h productivity [36]. This finding establishes a new benchmark for shikimate production in *E. coli*, recording the highest titer and productivity to date. Likewise, Zhang et al. devised an efficient strategy for producing d-allulose using a catalyst developed by assembling the d-allulose synthetic cascade in the *E. coli* envelope, eliminating the need for costly purified enzymes and by-products. Process optimization strikingly improved the d-allulose titer by 1,500%, and the production was scaled up to a 3-L scale, yielding 5.67 g L^−1^ d-allulose [37]. This approach holds promise for sustainable d-allulose production. *E. coli* is also amenable to genetic modification to produce MPL, a key vaccine adjuvant [38]. By removing specific gene clusters and overexpressing selected genes, a new strain, MW012, was created and was further modified into MW012/pWEPL, which produced two MPL species, with the more effective hexa-acylated MPL constituting 75% [38]. This innovative method offers a potentially more productive and cost-efficient MPL production route, with significant implications for vaccine development and wider biotechnological applications.

The phenolic compound 3,4-dihydroxycinnamic acid, commonly known as caffeic acid, is a valuable precursor for a vast array of several high-value compounds. Caffeic acid was efficiently produced in an *E. coli* strain, CA3, engineered to express tyrosine ammonia-lyase, *p*-coumaric acid 3-hydroxylase, and feedback-resistant chorismate mutase/prephenate dehydrogenase. Using this strategy, CA3, directly converted glucose into caffeic acid, yielding 1.58 g/L without the need for tyrosine supplementation. To further enhance the yield, 4-hydroxyphenylacetate 3-hydroxylase was introduced, resulting in an engineered strain, CA8, that produced 6.17 g/L of caffeic acid directly from glucose [39]. The developed *E. coli* strain holds promise as a framework strain for the biosynthesis of caffeic acid derivatives. In another study, both nonproteinogenic and proteinogenic amino acids have been successfully produced in engineered *E. coli* strains to efficiently produce AAA by modifying lysine biosynthesis and degradation pathways [40]. Notably, transgenic enzymes from other organisms, including *Saccharomyces cerevisiae* and *Rhodococcus erythropolis*, were introduced, which enhanced lysine supply while improving AAA efflux using a glutamate transporter from *Corynebacterium glutamicum*. The engineered strain achieved high AAA production levels of 2.94 g/L in flasks subjected to shaking and 5.64 g/L in bioreactors [40]. This method offers a sustainable and cost-effective alternative to chemical synthesis for industries such as pharmaceuticals, chemicals, and animal feed. Just as caffeic acid is very important industrially, l-arginine’s versatile properties, from enhancing nitric oxide production to promoting protein synthesis, make it an indispensable ingredient in various industrial applications, ranging from pharmaceutical manufacturing to food production. To improve l-arginine production, Nie et al. employed a key strategy of directly feeding *N*-acetylglutamate to engineered *E. coli*, bypassing the regulation and metabolic complexity of l-arginine biosynthesis [41]. Gene disruption (*argA*, *astA*, *speF*, *speB*, and *argR*) and operon overexpression (*argDGI* and *argCBH*) enabled high-yield l-arginine production (4 g/L) with a yield of 0.99 mol l-arginine/mol *N*-acetylglutamate in shake flask fermentation. Additionally, the co-production of l-arginine and pyruvate (4 g/L l-arginine and 11.3 g/L pyruvate) was achieved by improving the utilization of input carbon sources [41]. These findings have a far-reaching impact on industrial production and metabolic engineering. Similarly, LA, otherwise known as α-LA (ALA), is also another valuable substance widely used as a nutraceutical, supplement, and drug, with high industrial demand. Lennox-Hvenekilde et al. engineered an *E. coli* strain to overcome the challenge of toxicity associated with producing LA [42]. The pathway for LA production involves three key enzymes, all of which are toxic when overexpressed in *E. coli*. To overcome this toxicity, they identified enzymes from *Serratia liquefaciens*, exhibiting high catalytic efficiency and no cytotoxicity. The optimized strain produced 2.5 mg of free LA per g of glucose in minimal media and reached an LA titer of 87 mg/L of free LA after 48 h of fed-batch fermentation [42]. This titer is approximately 3,000-fold higher than formerly reported free LA titers and has implications for the industrial production of LA [42].

Erythritol, a sugar alcohol used as a sugar substitute, cannot be metabolized by humans [43]. Due to incomplete absorption in the body, some erythritol can reach the large intestine, potentially causing digestive issues such as bloating, gas, and diarrhea, especially in individuals who consume large amounts of erythritol. Recently, a strain of *E. coli* has been engineered to metabolize erythritol as its sole carbon source [43]. The genetic components involved in erythritol metabolism were elucidated, and the erythritol-binding transcriptional repressor and its target DNA region were studied. Transcriptome analysis reveals upregulation of carbohydrate metabolism genes, including transketolase (tktA and tktB) and transaldolase (talA and talB) genes. Overexpressing these genes led to a threefold increase in *E. coli* growth [43]. The genetically modified *E. coli* strains function as sensors for erythritol-containing soda soft drinks and exhibit the ability to proliferate in simulated intestinal fluid containing erythritol. These findings open possibilities for applications in synthetic biology, metabolic, and biomedical engineering. In a recent study, two heterologous pathways in *E. coli* have been constructed to produce 2-phenylethanol (2-PE) from glucose, where the Ehrlich pathway performed better and resulted in a higher titer [44]. The pathway was modified by deleting *feaB* and knocking out *crr* and *pykF* genes, which led to a 35% increase in titer. A plasmid-free strain was constructed with multiple ARO10 cassettes and overexpression of the yjgB gene, demonstrating exceptional performance in *de novo* 2-PE synthesis in *E. coli*. It achieved a yield of 0.076 g/g glucose and a productivity of 0.048 g/L/h, outperforming all previously reported values [44]. The resulting strain portrays the potential to produce higher titers with further fermentation optimization. Similarly, combinatorial metabolic engineering and tolerance evolving were employed to engineer the MG1655 *E. coli* strain to achieve a high 2′-FL production [45]. Precise regulation of gene expression in the 2′-FL synthesis pathway was achieved by employing single or multiple gene copies based on the identification of rate-limiting enzymes. This guided the elimination of competing branch pathway genes and the optimization of 2′-FL efflux protein SetA and fructose-1,6-bisphosphatase GlpX overexpression. This strain yielded an impressive 46.06 ± 1.28 g/L of 2′-FL in a 5-liter fermenter. Further refinement through adaptive laboratory evolution resulted in rpoC gene mutation that enhanced cell tolerance and boosted 2′-FL production to 61.06 ± 1.93 g/L. This established the highest productivity of 1.70 g/L/h among *E. coli* strains reported to date. Likewise, a genetically engineered *E. coli* strain produced HT, a valuable polyphenolic molecule [46]. The modified strain carries two plasmids that enhance the expression of key enzymes involved in HT production. The study showed that DOPA decarboxylase (DODC), alcohol dehydrogenases, monoamine oxidase, and glucose dehydrogenases are crucial for efficient HT biosynthesis. DODC from humans was found to be the most effective enzyme for HT production. Seven promoters were strategically integrated to enhance the expression of catalase, a crucial enzyme that catalyzes the decomposition of hydrogen peroxide (H_2_O_2_), a potentially harmful by-product of cellular metabolism. After screening and optimization, coexpression strains with enhanced HT production were obtained. This outcome has significant implications for the industrial production of HT. The optimized whole-cell biocatalyst exhibited remarkable performance, achieving a maximum titer of 4.84 g/L of HT and demonstrating an outstanding molar substrate conversion rate of over 77.5% [46]. This approach offers a more sustainable method for meeting the high demand for HT in various industries.

To find an efficient and cost-effective method for the bio-based industrial production of *cis*-3-HyPip, two enzymes, *Streptomyces malaysiensis* cyclodeaminase and StGetF (*Streptomyces* pipecolic acid hydroxylase), were screened for their ability to convert l-lysine into *cis*-3-HyPip [47]. They were expressed in *E. coli* W3110 Δ*suc*CD (a strain capable of producing α-ketoglutarate) and overexpressed an NAD(P)H oxidase to create an NAD + regeneration system. This enabled the efficient bioconversion of *cis*-3-HyPip from low-cost substrate l-lysine without the need for additional NAD + and α-ketoglutarate. The efficiency of the *cis*-3-HyPip biosynthetic pathway was also improved by optimizing multiple-enzyme expression and regulating transporter dynamics. The resultant strain, HP-13, achieved a remarkable production level of 78.4 g/L of *cis*-3-HyPip with a conversion rate of 78.9% [48], which is the highest reported so far. Likewise, successful whole-cell P450 biocatalysis using engineered *E. coli* and fine-tuned heme biosynthesis was recently reported [49]. Key strategies encompassed improving intracellular heme biosynthesis through synthetic gene expression and DNA-guided scaffolds, fine-tuning intracellular heme levels using mutated heme-sensitive biosensors and small regulatory RNA systems, and enhancing catalytic efficiencies of three P450s, namely, CYP105D7, BM3, and sca-2 [49]. The findings indicate that insufficient heme supply limits the activity of whole-cell P450 biocatalysis. This research has potential industrial applications, providing a strategy for developing whole-cell biocatalysts for chemical intermediates, drugs, and natural products. Overall, these achievements underscore the significance of *E. coli* in sustainable and efficient production processes. Furthermore, they offer valuable insights and methodologies, propelling the application of *E. coli* in sustainable and cost-effective industrial biotechnology.

### 1.2 Enzyme and metabolic engineering for the production of valued compounds in *S. cerevisiae* and other yeasts

*S. cerevisiae*, akin to *E. coli*, has been a foundation of recent advancements in protein engineering, enabling the efficient production of valuable substances. For instance, yeasts have been used to enhance the production of carotenoids and microbial fats [50,51,52]. Vitamin B12 is indisputably an essential material in many foods and pharmaceutical industries and is in high demand. A vitamin B12-dependent *S. cerevisiae* strain was developed by substituting the B12-independent methionine synthase gene with its B12-dependent counterpart from *E. coli*. Furthermore, a highly efficient bacterial flavodoxin/ferredoxin-NADP + reductase (Fpr-FldA) system was introduced and is essential for the *in vivo* reactivation of the MetH enzyme activity and yeast cell growth [53]. This engineered strain can only grow on methionine-free media when adenosylcobalamin or methylcobalamin is added. Notably, a heterological vitamin B12 transport system is not necessary for the uptake of cobalamin in these yeast cells. This result holds serious implications for industrial vitamin B12 production, as *S. cerevisiae* can be utilized as a host for heterological vitamin B12 production. Similarly, α-farnesene, a versatile natural sesquiterpene, finds applications in various industries, including fragrances, cosmetics, flavorings, insect repellents, pheromones, biofuels, pharmaceuticals, plastics, and agrochemicals. In recent research, an efficient α-farnesene-producing *S. cerevisiae* using metabolic engineering was created [54]. *Camellia sinensis* α-farnesene synthase (CsAFS) proved most effective, and the precursor supply was boosted by improving the mevalonate pathway. The introduction of CsAFS^W281C^ and a serine–lysine–isoleucine–lysine peptide tagged to the N-terminal further optimized production [54], yielding 2.8 g/L in shake-flask cultures and 28.3 g/L in bioreactor fermentation. The applications span across agriculture, fuel, and chemical industries. Fumaric acid is utilized as an acidulant in food and beverages, pharmaceuticals, resins, polymers, cosmetics, animal feed, and cleaning products. By disrupting the natural FUM1 gene and the introduction of genes for fumarase and malate transporter, a genetically modified *S. cerevisiae* that converts malic acid into fumaric acid was created [55]. The origin of the fumarase gene, the presence of the XYNSEC signal secretion peptide, and the maintenance of aerobic conditions during cultivation were all critical factors contributing to the successful outcome. Under aerobic conditions, the two strains ΔFUM1(ss)Ckr_fum + mae1 and ΔFUM1Ckr_fum + mae1 efficiently transformed extracellular malic acid into fumaric acid, achieving yields of 0.98 and 1.11 g/L, respectively.

Another industrially important chemical is Linalool, which is widely used in fragrances, flavors, perfumes, soaps, shampoos, and cleaning agents for its pleasant floral scent. Through protein engineering, modifications to the substrate-binding pocket of linalool synthase were made by introducing specific mutations (F447E and F447A). These alterations were employed to enhance the catalytic efficiency toward geranyl pyrophosphate. The strategy led to a substantial 2.2 and 1.9-fold increase in linalool production, respectively [56]. By compartmentalizing linalool synthesis in both the cytoplasm and peroxisomes of *S. cerevisiae*, a significant improvement in precursor molecule availability and optimized linalool production was achieved. The genetically enhanced strain achieved an impressive production level of 219.1 mg/L during shake-flask cultivation. Furthermore, in a 5-L capacity fed-batch fermentation, the diploid strain surpassed all previous records, reaching a linalool titer of 2.6 g/L, the highest reported production in the yeast system. These findings carry potential implications for the microbial manufacture of other monoterpenes and can be applied in industrial settings for sustainable and scalable linalool production. Glucaric acid’s biodegradability, non-toxicity, and potential to replace harmful substances make it an industrially relevant platform with applications in detergents, corrosion inhibitors, and pharmaceuticals. The fusion of MIOX4 and Udh enzymes, linked by the peptide (EA_3_K)_3_, in *S. cerevisiae* strain, GA16, enhanced the biosynthesis pathway of glucaric acid, achieving a 4.9 g/L titer value during shake flask fermentation [57]. Further engineering to regulate myo-inositol metabolism increased production, with strain GA-ZII producing 8.49 g/L in shake flask fermentation and 15.6 g/L in a 5-L bioreactor through fed-batch fermentation. Similarly, carminic acid, a natural colorant with enduring vibrancy and stability, finds diverse applications in food, cosmetics, pharmaceuticals, textiles, and art, driven by its sustainability. Through systematic pathway engineering in *S. cerevisiae*, a record carminic acid titer of 7580.9 μg/L was achieved in a 5-L capacity bioreactor [58]. This innovation opens up the possibility of environmentally friendly carminic acid production with higher yields and consistent quality, impacting the food and cosmetics industries. In the area of cosmetics and skincare, mycosporine-like amino acids (MAAs) are gaining traction as natural UV protectants, replacing synthetic sunscreens that may cause skin irritation or allergies. Their ability to absorb UV rays without harming the skin makes them a desirable choice. Recently, biosynthetic genes from cyanobacteria were integrated into *S. cerevisiae* to create a MAA-producing strain [59]. By introducing mysD genes, it was discovered that different MysD enzymes efficiently produce specific MAAs. MysD substrate specificity is determined by a 43-45 amino acid omega loop region. This research successfully demonstrates the production of MAAs in *S. cerevisiae*, including porphyra-334 and M2G, for the first time. In fermentation, the engineered strains achieved high titers of shinorine and porphyra-334, up to 1.53 and 1.21 g/L, respectively [59], offering a sustainable and environmentally friendly option for natural sunscreen development.

Apart from *S. cerevisiae*, other yeasts have applications in industrial bioengineering. For instance, recently, *Komagataella phaffii* (also known as *Pichia pastoris*), a methylotrophic yeast, was metabolically engineered for the overproduction of patchoulol [60]. Patchoulol holds industrial relevance in the fragrance and cosmetics industry, imparting a distinctive and enduring aroma to diverse products. The result showed that strain P6H_53_ produced 149.64 mg/L of patchoulol in shake-flask fermentation. In a 5-L capacity fermenter, P6H_53_ achieved a patchoulol yield of 2.47 g/L, and 21.48 mg/g DCW, in addition to a production rate of 283.25 mg/L/day. This research suggests the potential for industrial application and provides insights into high-yield patchoulol production in *K. phaffii* utilizing methanol as the source of carbon. Similarly, a dimorphic oleaginous, *Yarrowia lipolytica*, was recently metabolically engineered for improved application in the production of orcinol glucoside (OG). OG, known for its antidepressant activity, has industrial interests, primarily in the pharmaceuticals. This alteration in the *Yarrowia lipolytica* resulted in a 100-fold increase in OG production, yielding 43.46 g/L (0.84 g/g DCW) [61]. Finally, genome engineering of the yeast *Kluyveromyces marxianus* aimed to produce a high titer and yield of d-lactic acid under low pH without growth defects [62]. d-lactic acid, a versatile chiral organic compound, holds immense industrial potential in biodegradable plastics, food, pharmaceuticals, leather, dairy, and chemical industries, but cost-effective production remains an obstacle. Strategies used included genetic modification, optimized fermentation conditions, and resource utilization. Interestingly, the engineered strain, KMΔpdc1: ldhA, solely produced d-lactic acid without by-products. Aeration rate, pH, and temperature were optimized for a maximum d-lactic acid titer of 42.97 ± 0.48 g/L, obtained from glucose. Thermotolerance was also observed at 42°C. Sugarcane molasses proved to be a viable nutrient-less medium, yielding 66 g/L of d-lactic acid at 30°C. This engineered strain demonstrates scalability, cost-effectiveness, and potential for industrial production. Overall, the versatile yeast *S. cerevisiae* and other yeasts proved instrumental in industrial bioengineering, exemplified by the development of the vast array of valuable substances with scalable industrial applications.

### 1.3 Engineering other bacteria, fungi, and algae for the biosynthesis of specific compounds

*E. coli* is arguably the most prominent bacterium for industrial applications, as is *S. cerevisiae* for yeasts. However, other organisms, though not as prominent, also have spectacular applications in bioengineering with scalable industrial potential. Here, reference is made to the recent use of some of these other organisms to improve the production of valued substances. To overcome challenges in keratinase production and biomass generation hindering industrial use, a growth-dependent system in *Bacillus subtilis* was devised [63]. Utilizing quorum sensing and the P_*aprE*_ promoter, keratinase production was effectively delayed to promote host cell proliferation. Equilibrium between cell growth and keratinase production was optimized through copy number screening and core region mutations. The system’s efficiency was improved by the addition of 2% glucose during the early fermentation stage, significantly facilitating biomass accumulation. The successful implementation of this growth-dependent system enabled increased cell density from 8 to 15.6 and a rise in keratinase activity from 1,162 to 4,200 U·mL^−1^ [63]. The practical application of the system demonstrated feather waste conversion, resulting in the production of soluble keratins, amino acids, and polypeptides. *B. subtilis* was also useful in the recent strategy to enhance selenocysteine (SeCys) biosynthesis for efficient production of seleno-methylselenocysteine (SeMCys), which has applications in selenium supplementation [64]. Selenium supplementation was shown to have promising effects on critically ill patients by altering inflammatory markers and its antioxidant properties. Selenium influences the biosynthesis of proteins in cells, leading to differential protein expression, and induces lipid peroxidation [65,66,67,68,69,70]. Intracellular accumulation of SeCys was achieved by overexpressing serine O-acetyltransferase and optimizing the S-adenosylmethionine (SAM) synthetic pathway via enhancing SAM synthetase expression. SeMCys was successfully produced by expressing selenocysteine methyltransferase in a strain accumulating both SeCys and SAM. Integration of necessary genes yielded SeMCys production at 18.4 μg/L. This lays the foundation for further pathway engineering for large-scale SeMCys production, with significant implications for supplementation and pharmaceuticals in the industry. *Lactococcus lactis* metabolic engineering has demonstrated the first successful extracellular production of 4,6 and 4,3 α-glucanotransferases [71]. The enzymes have Generally Recognized as Safe status and are potentially useful for industrial applications, particularly in starch retrogradation delay and reduction of glycaemic index.

In *Clostridium cellulolyticum*, genetic engineering, and CRISPR-Cas9n were employed to enhance cellulose degradation. Cellulose degradation, a vital natural process facilitated by microorganisms, possesses significant industrial importance. This process enables the production of biofuels, paper, textiles, biochemicals, and solutions for wastewater treatment. By integrating a more efficient β-glucosidase and disrupting the expression of lactate dehydrogenase (ldh), the manipulated strain showed a 7.4-fold enhancement in β-glucosidase activity and a 70% reduction in lactate production [72]. It exhibited a 12% improved degradation of cellulose and a 32% higher ethanol production rate compared to the wild-type. The study identifies ldh as a potential locus for heterologous expression, proving the effectiveness of this strategy. Similarly, in different research aimed at enhancing cellulose degradation, genetically engineered *Lactobacillus lactis* strains, NZ9000s, were created to express and produce high-efficiency β-glucosidase enzymes. These enzymes basically comprised of three subunits (BglA, BglB and Bgl) of β-glucosidase found in yak rumen microorganisms [73]. The engineered strains showed impressive efficient hydrolysis of cellulose through the production of β-glucosidase enzymes [73].

β-Carotene is important industrially as a provitamin A source, and is widely used in food, cosmetics, pharmaceuticals, and animal feed. The filamentous fungus *Trichoderma reesei* was recently engineered to simultaneously produce cellulase and β-carotene [74], offering dual benefits for industrial applications. The approach involved overexpressing four key enzymes, including CarRP, HMG1, GGS1/CrtE, and CarB, resulting in β-carotene production, attaining 218.8 mg/L titer in flask culture and 286.63 mg/L in a jar system, along with cellulase production of 22.33 IU/mL [74]. Similarly, glucoamylase, a vital enzyme for saccharification of starch, is mainly produced by mesophilic fungi in the biofuel and food industries. The thermophilic fungi, *Myceliophthora thermophila* was recently engineered, with improved AsCas12a variants to develop a platform for the high production of glucoamylase in *M. thermophila* [75]. Gene deletions created the strain MtGM12, with a significant increase in glucoamylase secretion and activity compared to the wild-type strain. In agriculture, physcion is potentially an ecofriendly fungicidal alternative for pest and disease management in crops. The enzyme O-methyltransferase enzyme AcOMT was recently identified and employed to *de novo* biosynthesize the natural fungicide physcion, by optimizing the biosynthetic pathway in *Aspergillus nidulans*. Basically, emodin biosynthesis was used as a probe to identify AcOMT, which is capable of converting emodin into physcion. The introduction of AcOMT into *A. nidulans*, along with expressed decarboxylase genes, achieved a physcion titer of 64.6 mg/L in shake-flask fermentation [76]. The biosynthetic pathway was optimized by expressing decarboxylase genes from different fungi.

Ketocarotenoid accumulation in the green algae *Chlamydomonas reinhardtii* holds significant industrial relevance by enabling the production of valuable compounds. Such compounds include astaxanthin, improving tolerance to highlight conditions, offering the potential for genetic modification to enhance carotenoid content, and contributing to the generation of bioactive compounds with diverse applications. This positions the microalga as a promising candidate for industrial use. Recently, *C. reinhardtii* was metabolically engineered for efficient ketocarotenoid accumulation. The strategies include a systematic screening of synthetic transgene designs that target carotenoid pathway enzymes, the identification of phytoene synthase (PSY/crtB) as a limiting factor, and the enhancement of β-carotene hydroxylase (CHYB) activity to promote the accumulation of engineered astaxanthin. The implementation of a unified BKT, crtB, and CHYB expression strategy yielded a 4-fold enhancement in volumetric astaxanthin production compared to preceding studies [77]. Overall, *B. subtilis*, *C. cellulolyticum*, *L. lactis*, *T. reesei*, *M. thermophila*, and *C. reinhardtii* are just a few examples of organisms that, apart from *E. coli* and *S. cerevisiae*, demonstrate the diversity and applicability of bioengineering efforts to enhance the production of valuable substances.

## 2 Enzyme engineering for enhanced thermostability

There are many parameters that are crucial to the efficiency of enzymes used for industrial applications, including pH, solvent, temperature, substrate concentration, enzyme concentration, cofactors, and coenzymes. However, the thermostability of enzymes is an exceptionally crucial necessity for enzyme efficiency, as many industrial processes often require high temperatures. Extremophiles, organisms thriving in extreme environments, have been discovered to produce enzymes with high stability against heat, salts, and pH, providing valuable insights for designing stable enzymes. Recently, researchers have discovered ways to improve the thermostability of enzymes. For instance, through rational engineering, two mutations (D28N and D116N) that enhanced the thermostability (6.1 and 9.2°C increase, respectively) and activity of a metalloprotease enzyme were identified using molecular dynamics simulation [78]. The mutants demonstrated increased stability, higher melting temperatures, longer half-lives (1.07- and 1.8-fold higher) at high temperatures, and improved synthetic activity. Increased interactions due to hydrogen bonds and regional hydration shells contributed to the enhanced thermostability. Similarly, isothermal compressibility (*β*_T_) perturbation engineering (ICPE) was employed in engineering a variant of T1 lipase with enhanced thermostability and activity [79]. ICPE is a strategy that aims to understand the stability-activity trade-off in enzymes and develop variants that exhibit both high activity and stability. The most promising variant, A186L/L188M/A190Y, exhibited a high melting temperature (*T*_m_) of 78.70°C, a catalytic activity of 474.04 U/mg, and a 73.33% increase in resistance to dimethyl sulfoxide compared to the wild-type [79]. *Bacillus amyloliquefaciens* alpha-amylase is widely used in food and cleaning industries and was enhanced for thermostability by introducing multipoint mutations. The resultant mutant enzyme, MuBAA (D28E/V118A/S187D/K370 N), demonstrated superior thermostability and a 99.1% increase in specific activity (206.61 U/mg) compared to the wild-type [80]. Its enhanced thermostability was attributed to structural changes, including stabilization of a loop region in domain B and additional intramolecular interactions in domains A and B.

Two engineering approaches were used to improve the thermostability of polyethylene terephthalate (PET) hydrolase enzymes, which are crucial for efficient and cost-effective PET degradation and recycling. The first approach involved covalently cyclizing *Ideonella sakaiensis* PETase (IsPETase) using SpyCatcher-SpyTag technology, which increased thermostability but reduced PET substrate turnover [81]. The second approach utilized a GFP-nanobody fusion protein (vGFP) as a scaffold, yielding a construct with an 80°C melting temperature, which further improved to 85°C when a thermostable PETase variant (FAST PETase) was incorporated. This is the highest reported thermostability for an engineered IsPETase-derived PET hydrolase. Notably, the vGFP scaffold-enhanced thermostability did not compromise PET hydrolase activity, indicating that scaffold choice impacts both thermostability and enzymatic activity. These findings have industrial implications, as thermostable PET hydrolase enzymes can improve PET degradation efficiency and robustness. This reduces enzyme quantities needed for recycling, contributing to more sustainable and environmentally friendly PET recycling practices. Recently, also, Protein Repair One-Stop Shop algorithm was employed to engineer a thermostable variant of mFMO, a flavin-containing monooxygenase (FMO). By introducing 8 to 28 mutations, seven mutant variants of mFMO were generated, resulting in increased melting temperatures ranging from 4.7 to 9.0°C [82]. The most thermostable variant, mFMO_20, exhibited a crystal structure with four new stabilizing interhelical salt bridges involving mutated residues. Notably, mFMO_20 outperformed the native mFMO in reducing trimethylamine (TMA) levels in a salmon protein hydrolysate at industrially relevant temperatures. By enzymatically converting TMA into the odorless trimethylamine N-oxide, the engineered mFMO variant presents a promising solution to address the issue of fish-like odor in marine by-product-derived protein hydrolysates. Similarly, the thermostability of d-carbamoylase from *Nitratireductor indicus* (NiHyuC) was enhanced through ancestral sequence reconstruction and consensus sequence analysis [83]. Iterative combinatorial mutagenesis generated mutant enzyme M4Th3 (S202P/E208D/R277L), which exhibited significantly increased thermostability compared to M4 [83]. M4Th3 has a half-life of 36.5 h at 40°C, while M4 only lasts 1.3 h. M4Th3 also achieves higher conversion rates, converting 96.4% of *N*-carbamoyl-d-tryptophan to d-tryptophan after 12 h at 40°C, compared to M4’s 64.1%. Molecular dynamics simulations elucidate that the E208D mutation enhances hydrophobicity, while the R277L mutation improves the protein’s homodimeric structure stability. These findings have significant implications in the industrial “hydantoinase process” for synthesizing optically pure amino acids.

The thermal stability of sucrose isomerase from *Erwinia rhapontici* NX-5 was increased by a combination of strategies. Nineteen high B-value amino acid residues were identified and subjected to site-directed mutagenesis. The modified enzyme mutants (K174Q, L202E, and K174Q/L202E) were expressed and characterized in *Pichia pastoris* X33, demonstrating improved thermal stability with optimal temperatures increasing by 5°C and half-lives increasing 2.21, 1.73, and 2.89 times, respectively [84]. The mutant enzymes also showed increased activity by 20.3% to 25.3% and decreased *K*_m_ values by 5.1, 7.9, and 9.4%, resulting in up to 16% higher catalytic efficiency. The comprehensive strategy used in this study can be applied to enhance the thermal stability of other enzymes. In a similar strategy, the thermostability of L-asparaginase (ASNase) was enhanced by mutating three key residues (Gly97, Asn159, and Glu249) to TYQ. This resulted in a triple mutant enzyme that was significantly more thermostable than the wild-type enzyme [85]. Molecular dynamics and structural analysis revealed that the added hydrogen bonds, increased hydrophobic interactions, and advantageous electrostatic potential contributed to the enzyme’s increased rigidity and thermostability. Finally, to improve UDP-glucose synthesis, the thermostability of sucrose synthase (Susy) from *Nitrosospira multiformis* was improved using automated prediction and greedy accumulation of beneficial mutations. The mutant Susy (M4) exhibited 27-fold increased T1/2 value at 55°C, allowing for UDP-glucose synthesis at 37 g/L/h [86]. Molecular dynamics simulations revealed that newly formed interfaces, particularly involving residue Trp162, strengthened subunit interaction. Overall, enhancing enzyme thermostability through molecular manipulation of inter-residue interactions holds significant promise for their industrial applications.

## 3 Future perspectives

Precision engineering and artificial intelligence can enhance enzyme activity and predictability [25,87]. For instance, a novel AI tool, referred to as CLEAN, surpasses top-tier tools in terms of precision, dependability, and responsiveness [88]. This tool utilizes the language inherent in proteins to predict their activity. CLEAN has the capability to forecast the roles of enzymes that have not been previously defined. It can rectify inaccuracies in enzyme labeling and accurately pinpoint enzymes that possess dual or multiple functions. Furthermore, machine learning can utilize the amino acid sequences of enzymes that are structurally similar but functionally different as input data, enabling efficient progression toward the desired function. This could potentially offer a fresh, fundamental understanding of the specificity between enzymes and substrates.

Furthermore, the study of microbial communities can lead to the development of efficient enzyme consortia. This, combined with the integration of enzymes with nanomaterials and synthetic biology, can unlock new functionalities and refine production methods. The focus of future engineering will be on enhancing enzyme tolerance and compatibility with unconventional substrates, thereby transforming waste into valuable products. Interdisciplinary collaboration will be pivotal in optimizing enzyme structure-function relationships and refining production processes. Tailored enzyme properties, directed evolution, and computational approaches will broaden the enzyme repertoire to cater to diverse industrial needs. The implementation of multi-enzyme cascades, immobilization techniques, novel reactor designs, and engineered enzymes will streamline complex synthesis pathways, enable continuous processes, and facilitate bioremediation. Enzyme-based biosensors will offer real-time process monitoring, contributing to the production of sustainable materials and biofuels. In the healthcare sector, the development of personalized therapeutics based on engineered enzymes will revolutionize treatment approaches. Collectively, these advancements will align industrial practices with the principles of sustainability and efficiency, marking a new era in protein engineering.

Protein engineering has indeed catalyzed a revolution in industrial applications, yet it is not devoid of challenges when it comes to the utilization of these modified proteins. A primary obstacle is the substantial cost incurred due to the complex procedures and specialized equipment required. Another concern is the stability of these engineered proteins; they may lose functionality if environmental conditions such as temperature or pH levels fluctuate. Additionally, there is the potential for unforeseen consequences. These proteins could interact with other molecules unpredictably, which might lead to detrimental effects. The process of obtaining regulatory approval for these proteins can be protracted and rigorous, especially for applications in the food and pharmaceutical industries. Ethical considerations also come into play, as the employment of GMOs in protein engineering is a contentious issue and often encounters public resistance. It is also crucial to note that safety concerns, legal regulations, and societal biases present distinct challenges. Independent experiments on microorganisms, which are a relatively recent development, could potentially provoke strong responses from governmental bodies. Despite this, many regulations continue to prohibit even the most basic genetic manipulations, such as bacterial transformation with a plasmid, outside of designated research facilities. Nevertheless, despite these obstacles, the benefits conferred by protein engineering frequently outweigh the disadvantages, rendering it a field ripe for future investigation and advancement.
